# NK cells improve control of friend virus infection in mice persistently infected with murine cytomegalovirus

**DOI:** 10.1186/1742-4690-10-58

**Published:** 2013-06-05

**Authors:** Sandra Francois, Jing Peng, Tatjana Schwarz, Janine Duppach, Kathrin Gibbert, Ulf Dittmer, Anke RM Kraft

**Affiliations:** 1 Institute for Virology of the University Hospital in Essen, University of Duisburg-Essen, Essen, Germany; 2 Current address: Department of Gastroenterology, Hepatology and Endocrinology, Hannover Medical School, Hannover, Germany

**Keywords:** Superinfection, Heterologous immunity, mCMV, Friend virus, NK cells, HIV, CMV

## Abstract

**Background:**

Co-infection of HIV patients with cytomegalovirus (CMV) is associated with enhanced AIDS progression and CMV end-organ diseases. On the other hand, persistent CMV infection has recently been shown to decrease tumor relapse and protect against lethal bacterial infection. The influence of persistent CMV on the outcome of an acute retroviral superinfection is still unknown.

**Results:**

Here we show that a persistent murine CMV (mCMV) infection surprisingly confers higher resistance to a primary Friend retrovirus infection (FV) of mice. Decreased FV titers and augmented FV-specific CD8 T-cell responses were found in mCMV infected mice during primary FV superinfection. NK cells produced higher amounts of IFNgamma after FV infection of persistently mCMV infected mice suggesting that these cells were involved in the ‘protective’ effect. Depletion of NK1.1^+^ cells or neutralization of IFNgamma during FV superinfection abrogated the mCMV-mediated effect.

**Conclusion:**

Our data demonstrate for the first time that a persistent CMV infection induces long-lasting NK cell responses that can enhance immunity to primary retroviral infections. To our knowledge, studies investigating primary HIV infection have not analyzed the role of the CMV seropositivity in these patients. Our observations suggest that NK cells in CMV seropositive individuals might contribute to the control of primary HIV infection.

## Background

Approximately 34 million people worldwide are currently infected with HIV and approximately 2.5 million new infections occur per year. Co-infections of HIV infected individuals with unrelated viruses like human cytomegalovirus (CMV) are associated with AIDS progression and CMV end-organ diseases (CMV-EOD) [1]. In a natural course of CMV/HIV co-infection, CMV-EOD occurs predominantly in HIV patients with low CD4 T cell counts [2-5]. CMV is a very common viral infection and approximately 40-80% of the human population worldwide is CMV seropositive. In an immunocompetent individual a primary CMV infection is mostly asymptomatic and during latency CMV is well controlled by the immune system. CMV seropositivity in HIV infected patients or transplant recipients, as well as presence of CMV-infected cells in transplant organs, has been associated with poor prognosis, i.e. AIDS progression and transplant rejection [6-8]. However, these studies are mainly describing patients in the late phase of HIV infection when immunodeficiency starts to develop, but very little is known about acute HIV infection of CMV seropositive individuals. Recent publications have shown that persistent CMV infection can be beneficial for the host [9,10]; the group of Elmaagacli et al. showed that an early ‘controlled’ CMV replication reduced leukemia relapse in acute myeloid leukemia patients who underwent allogeneic stem cell transplantation [9]. Along with this finding, Barton et al. described that during persistent infection with murine cytomegalovirus (mCMV) or murine gammaherpesvirus 68 (γHV68) mice are protected against lethal bacterial infections by prolonged macrophage activation and IFNγ secretion [10]. To our knowledge nothing is known about the influence of a persistent CMV infection on a primary HIV infection. Based on the findings that CMV can be helpful for the host to control tumor relapse and bacterial superinfections it would be important to investigate the influence of a persistent CMV infection on the outcome of a primary retroviral infection.

Therefore, we established a mouse model to mimic a retroviral superinfection in a persistently CMV infected host. We used the well-established Friend virus (FV) as a model for retroviral infection and mCMV which has been used as a model to study CMV immunobiology and pathogenesis [11,12]. Similar to CMV, infectious mCMV can be isolated from infected mice over several months indicating a persistent infection. Additionally, strong NK cell and T cell responses are detectable in these mice [13]. Recently, it has been shown that NK cells are modulated during mCMV infection and memory-like NK cell population developed [14]. During acute mCMV infection the mCMV-specific protein m157 binds to the Ly49H receptor of NK cells and induces a proliferation of this subpopulation [15,16]. Friend virus (FV) is a retroviral complex comprised of two components, the replication-competent non-pathogenic helper virus called Friend murine leukemia virus and the replication-defective pathogenic spleen focus-forming virus [17]. In adult C57BL/6 mice a primary FV infection induces a transient splenomegaly [18]. During the acute phase of infection, virus-specific CD8 T cells are important for controlling viral replication, but NK and CD4 T cells are not significantly contributing to keep the infection in check [19,20].

Here we show for the first time that a persistent mCMV infection significantly reduced viral load during acute retroviral infection and demonstrate that mCMV induced NK cell and IFNγ responses augmented anti-viral T cell responses *in vivo*.

## Results

### Viral loads during primary FV infection were reduced in mice persistently infected with mCMV

**Figure 1:**
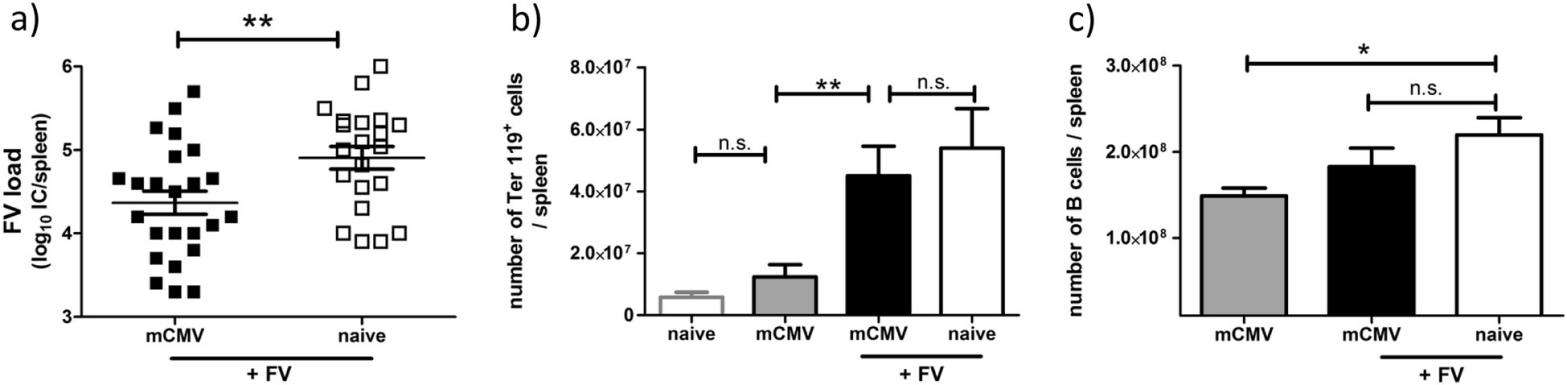
**mCMV persistence resulted in significantly decreased viral loads after FV superinfection.** Naïve or persistently mCMV infected mice (day 35 post mCMV infection) were infected with FV. At day 10 post FV infection **a**) FV titers and **b**,**c**) FV-specific target cells (Ter119^+^ erythrocyte precursor cells, CD19^+^ B220^+^ B cells) were determined in spleens. Statistical analysis **a**) unpaired student *t*-test, data pooled from 6 independent experiments, **b**) one-way ANOVA non-parametic Kruskal-Wallis test, data pooled from 3–4 independent experiments with 4–19 mice per group, **c**) one-way ANOVA Bonferroni’s multiple comparison test, data pooled from 2 independent experiments with 9 mice per group. Statistically significant differences between the groups are indicated by n.s. no significant differences, * p < 0.05, ** for p < 0.001.

To study the influence of a persistent mCMV infection during primary FV infection, mice were infected i.p. with mCMV. In the persistent phase of mCMV infection (5–10 weeks post infection), mice were superinfected i.v. with FV. At this time point mCMV was detectable by plaque assay in livers and salivary glands (Additional file 1: Figure S1) indicating mice were in the persistent phase of mCMV infection. As a control, age-matched naïve mice were infected with FV only. At day 10 after FV infection the acute FV load was determined in spleens (Figure 1a). A significant 4-fold reduction of FV titers was detected in the superinfected mice compared to mice infected with FV alone (Figure 1a). This result suggests that a persistent mCMV infection might have a beneficial effect on the clearance and disease progression of a primary retroviral infection.

During the acute phase of FV infection, there is an increase in the number of erythrocyte precursor cells (Ter119^+^), which represent the most important target cells of FV [21]. We therefore investigated if persistent mCMV infection interfered with the FV-induced expansion of Ter119^+^ target cells. However, we found no difference in number of Ter119^+^ cells in FV infected naïve and persistently mCMV infected mice (Figure 1b). Moreover, the numbers of CD19^+^ B cells, which also get infected by FV [21], were not different (Figure 1c). These data suggest that the reduced FV titers found in persistently mCMV infected mice were not a result of an impaired expansion of FV target cells.

### mCMV persistence augmented the FV-specific CD8 T cell response

**Figure 2:**
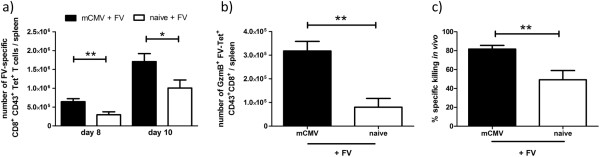
**mCMV persistence resulted in an augmented FV-specific CD8 T cell response. a**) Absolute numbers of FV-specific CD8 T cells were determined at day 8 and 10 by tetramer staining in spleens of FV infected naïve and persistently mCMV infected mice. Data are pooled from at least 2 (day 10) and 4 (day 8) independent experiments with 7–14 mice per group. **b**) Absolute numbers of granzyme B^+^ FV-specific CD8 T cells were determined at day 8 post FV infection in spleens of naïve (white bar) and persistently mCMV infected (black bar) mice. Data shown are 1 representative experiment out of 4 with 4 mice per group. **c**) *In vivo* cytotoxicity assay was performed as described in the material and methods section. Data shown are from spleens of 2 independent experiments with 7–8 mice per group. Statistical analysis: unpaired *t*-test. Statistically significant differences between the groups are indicated by * p < 0.05, ** for p < 0.001.

To determine if the reduced FV titers in persistently mCMV-infected mice were due to enhanced FV-specific immune responses, we analyzed the number of FV-specific CD8 T cells by tetramer staining at day 8 and 10 post FV infection (Figure 2a). Significantly increased numbers of activated (CD43^+^) FV-specific CD8 T cells were found in FV superinfected compared to only FV infected mice (Figure 2a). These differences were determined at both 8 and 10 days post FV infection, indicating that the kinetic and the magnitude of the FV-specific CD8 T cell response might be influenced by persistent mCMV. In addition to an increased magnitude of FV-specific CD8 T cell responses in superinfected mice, these cells were functionally augmented by superinfection as well. First, a significant 3.5-fold increased number of granzyme B^+^ FV-specific CD8^+^ tetramer^+^ T cells was detected at day 8 p.i. in FV superinfected compared to only FV infected mice (Figure 2b). To confirm that the FV-specific CD8 T cells expressing granzyme B were cytotoxic *in vivo*, we performed an *in vivo* cytotoxicity assay. Splenocytes from a naïve CD45.1^+^ mouse were loaded with the same viral peptide recognized by the tetramer^+^ CD8^+^ T cells and stained with CFSE. These labeled splenocytes together with unlabeled, control CD45.1^+^ splenocytes (without peptide) were injected i.v. into CD45.2^+^ naïve or persistently mCMV infected mice at day 8 after FV infection. In superinfected mice an average of 82% of the peptide-loaded target cells were eliminated within 2 hours compared to a killing of 49% in only FV infected mice (Figure 2c). These data show that a persistent mCMV infection resulted in increased numbers of functional FV-specific CD8 T cells at day 8 of FV infection of persistently mCMV infected compared to naïve controls.

### NK cells and IFNγ in persistently mCMV infected mice contributed to enhanced FV-specific T cell responses

**Figure 3:**
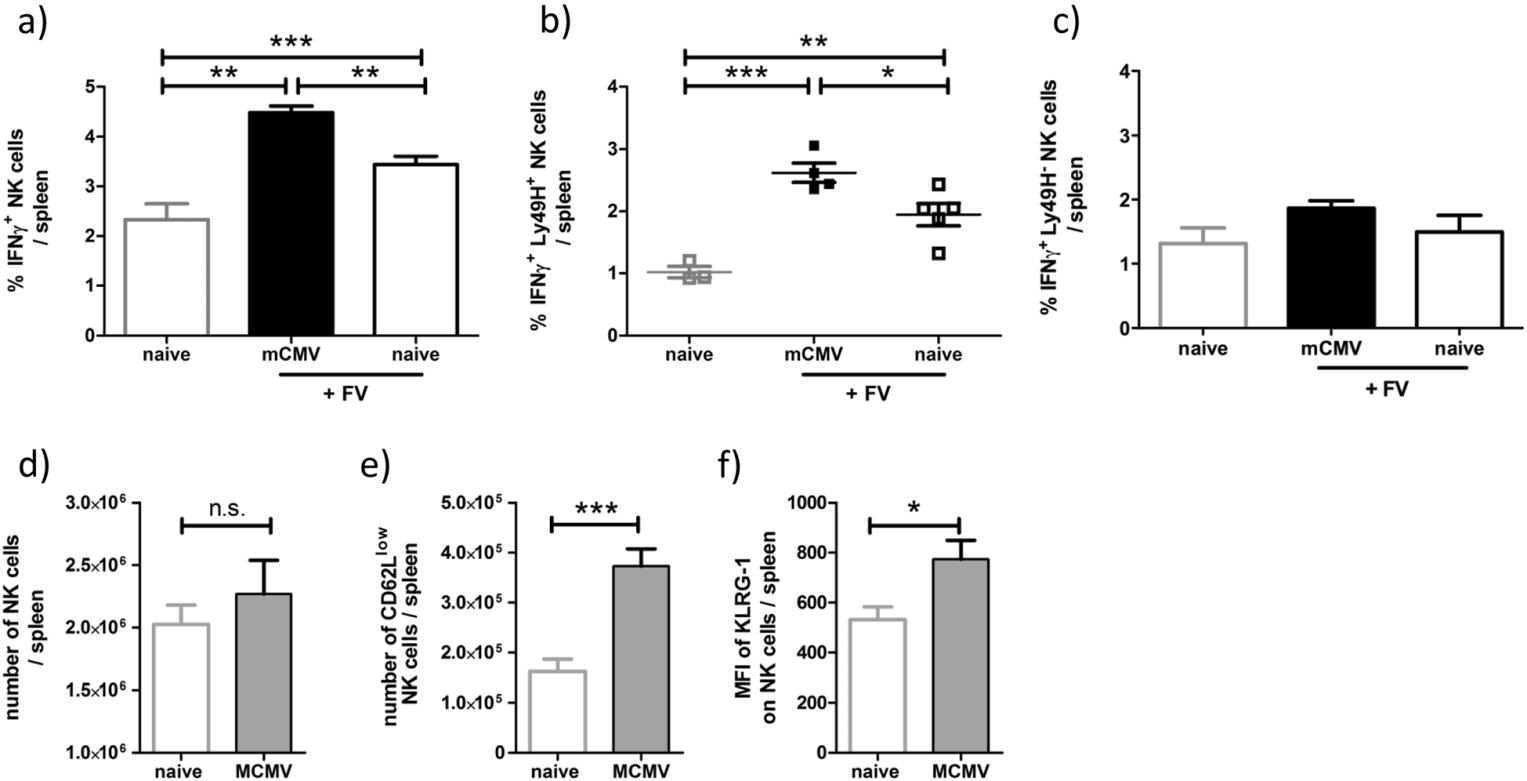
**Enhanced numbers of NK cells were determined in persistently mCMV infected compared to naïve mice.** IFNγ production by **a**) CD3^-^, DX5^+^, NK1.1^+^, **b**) Ly49H^+^ CD3^-^, DX5^+^ NK1.1^+^ and **c**) Ly49H^-^ CD3^-^, DX5^+^, NK1.1^+^ cells were determined in spleens of naïve or day 4.5 post FV infection in naïve (white bar) and persistently mCMV (black bar) infected mice. **d-f**) NK cells were characterized in the spleen of naïve and persistently mCMV infected mice. **d**) Absolute numbers of NK cells were determined by gating of NK1.1^+^ DX5^+^ CD3^-^ cells. Absolute numbers of CD62L^low^ (**e**) and MFI of KLRG-1 (**f**) gated on NK1.1^+^ DX5^+^ CD3^-^ cells were analyzed between the different groups. Data are pooled from **d**) 4–5 independent experiments with 9–16 mice per group, **e**,**f**) 3 independent experiments with 8–16 mice per group. Statistical analysis: unpaired *t*-test. Statistically significant differences between the groups are indicated by * p < 0.05, ** for p < 0.001, *** for p < 0.0001.

During acute CMV infection, NK cells are activated, undergo clonal expansion, and generate long-lived memory cells [10,22]. Mice persistently infected with γHV68 were protected against lethal challenge with *Listeria monocytogenes* due to enhanced IFNγ levels in the sera and prolonged macrophage activation [10]. Similar protection against *Listeria* infection was found in mice persistently infected with mCMV [10]. In order to test whether IFNγ and NK cell activity triggered by persistent mCMV infection contribute to enhanced FV-specific CD8 T cell responses, we first assessed IFNγ levels in the sera of naïve compared to persistently mCMV infected mice. At the time of FV superinfections (around 35 days post mCMV infection) enhanced IFNγ levels were detectable only in a few persistently mCMV infected mice and the overall difference between naïve and mCMV infected mice was not significant (data not shown). Similarly, one day and 4.5 days post FV superinfection only marginal concentrations of serum IFNγ were found in persistently mCMV infected mice (data not shown). However, at 4.5 days post superinfection a greater proportion of NK cells from mCMV infected mice were producing intracellular IFNγ relative to controls (Figure 3a,b). Notably, the Ly49H^+^ NK cells, which have been shown to expand during mCMV infection due to interaction with the mCMV protein m157 [23], were the main producers of IFNγ in superinfected mice (Figure 3b), whereas no significant difference in IFNγ production was detectable in Ly49H^-^ NK cells from mice of the two groups (Figure 3c). There were no differences in the total number of NK cells present in naïve mice and mice persistently infected with mCMV (Figure 3d), but there were statistically significantly differences in the phenotype of splenic NK cells. Specifically, persistent mCMV infection was associated with increased numbers of CD62L^low^ NK cells and elevated expression of KLRG-1 on NK cells (Figure 3e,f).

**Figure 4:**
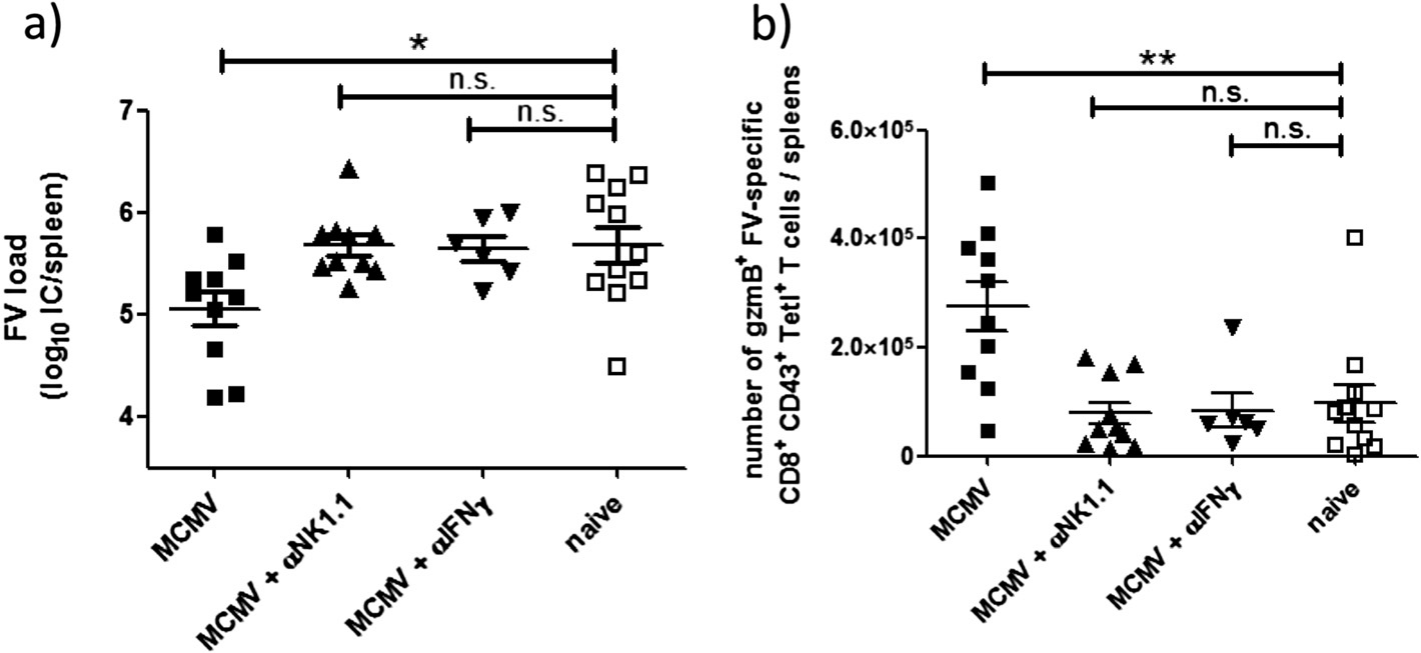
**NK cells and IFNγ in persistently mCMV infected mice contributed to reduced FV titers and enhanced FV-specific CD8 T cell responses.** Depletion of NK1.1^+^ cells and blockage of IFNγ was performed in persistently mCMV infected mice at day −1, 2 and 4 post FV infections. As control naïve (white dots) and persistently mCMV infected (black dots) mice were superinfected with FV. FV loads were detected by an infectious center assay (**a**) and functional FV-specific CD8 T cells were determined by granzyme B expression gated on tetramer + CD8 T cells (**b**) at day 8 post FV infection. Data shown are pooled from 2–4 independent experiments. Statistical analysis: one-way ANOVA (Dunett’s multiple comparison test). Statistically significant differences between the groups are indicated by n.s. no significant differences, * p < 0.05, ** for p < 0.001.

To further investigate whether the alterations in NK cell phenotype and function associated with persistent mCMV infection may contribute to the enhanced antiviral CD8 T cell responses observed during FV superinfection, NK1.1^+^ cell depletions were performed prior to and during primary FV infection of persistently mCMV infected mice. In a separate experiment, we administered antibodies to block IFNγ during FV superinfection in mCMV infected mice. Anti-IFNγ or anti-NK1.1 antibodies were injected i.p. at day −1, 2 and 4 of FV infection of naïve or persistently mCMV infected mice. At day 8 post FV infection, FV titers and FV-specific CD8 T cell responses were determined in spleens (Figure 4a, b). As seen previously, mice persistently infected with mCMV had significantly lower FV loads compared to mice infected with FV alone (Figure 4a, Figure 1a). Either depletion of NK1.1^+^ cells or blockade of IFNγ during acute FV infection of persistently mCMV infected mice resulted in FV loads comparable to those in mice infected with FV alone, indicating that NK1.1^+^ cells and IFNγ were involved in the mCMV-induced reduction of FV replication (Figure 4a). Since the FV-specific CD8 T cell response influences the FV load during acute infection, we quantified the FV-specific CD8 T cells after NK1.1^+^ cell depletion or IFNγ blockade in persistently mCMV infected mice (Figure 4b). The significantly enhanced number of granzyme B producing FV-specific CD8 T cells detected in persistently mCMV infected mice after FV infection was completely abrogated if NK1.1^+^ cells (NK and NKT cells) were depleted or IFNγ was blocked by antibodies. The number of GzmB^+^ FV-specific CD8 T cells in persistently mCMV infected mice receiving α-NK1.1 or α-IFNγ antibodies were comparable to only FV infected mice (Figure 4b). As shown before, NK cells [19] and IFNγ [24] have no direct effect on FV replication and FV-specific CD8 T cell responses between week one and two post FV infection of naïve mice (Additional file 2: Figure S2a-d).

## Discussion

Our data demonstrate for the first time that a persistent mCMV infection can be beneficial during a primary retroviral infection by augmenting anti-retroviral CD8 T cell immunity that resulted in decreasing FV loads in superinfected mice.

Previous reports demonstrated that mice persistently infected with γHV68 or mCMV were protected against superinfections with lethal doses of either *Listeria monocytogenes* or *Yersinia pestis *[10]. These effects were mediated by systemic activation of macrophages and prolonged IFNγ production in γHV68-infected mice. We were unable to detect prolonged macrophage activation (F4/80^+^, MHC classII^+^) in the spleen or sustained IFNγ secretion in the sera of mice persistently infected with mCMV (data not shown). A possible explanation is that a persistent γHV68 infection triggers a higher expression of IFNγ than the persistent mCMV infection. However, we were able to detect significant differences in the expression of IFNγ in NK cells between naïve and persistently mCMV infected mice early after FV infection. Moreover, blocking IFNγ during FV superinfection of mCMV infected mice resulted in enhanced FV titers and reduced FV-specific CD8 T cell responses, indicating that IFNγ was important in the mCMV-mediated effects. Depletion of NK1.1^+^ cells resulted in the same effect as IFNγ blockage. Our NK1.1^+^ depletion protocol depleted NK cells as well as NKT cells, but no effect on γδ^+^ and αβ^+^ CD8 T cells was found (data not shown). Therefore the loss of NKT cells might also contribute to the effect described after NK1.1^+^ depletion. However, our data suggest that persistent mCMV infection mainly modulated the NK cell response that resulted in significantly enhanced numbers of IFNγ^+^ NK cells at day 4.5 post FV infection, which might positively influenced the FV-specific CD8 T cell response.

The influence of cytokines on CD8 T cell priming differs depending on the time-point and amount of cytokine secretion during T cell priming. For example IL-2 or type I IFN can enhance or inhibit T cell responses depending on the time-point after virus infection [25-28]. Virus-specific CD8 T cell numbers are markedly reduced in IFNγ^−/−^ and IFNγR^−/−^ mice after LCMV infection [29] suggesting that IFNγ can be necessary for an effective primary anti-viral CD8 T cell response. However, it has also been shown that IFNγ can have a negative impact on virus-specific CD8 T cells [30]. In this study, a co-infection of FV together with the lactate dehydrogenase-elevating virus (LDV) resulted in a reduced FV-specific CD8 T cell response mediated by the strong LDV-dependent IFNγ production. Duley et al. reported that high concentrations of serum IFNγ were detected only immediately after infection (1 day post infection), which inhibited CD8 T cell priming. In contrast, high IFNγ serum concentrations were not found in our study, but intracellular IFNγ expression by splenic NK cells was detected starting at day 4.5 post FV infection of persistently mCMV infected mice. These results suggest that the timing and the strength of the IFNγ response may be critical for its effect on CD8 T cell priming in FV infection. Our data is supported by a previous study showing that secretion of IFNγ by activated NK cells can facilitate the priming of tumor-specific CD8 T cells [31].

Recent studies demonstrated that superinfections of β-herpesvirus infected mice with West Nile virus (WNV) or LCMV did not influence the outcome of the second infection [10,32,33]. There were no differences in the survival of naïve and γHV68-infected mice after WNV superinfection [10]. Our current results may be different from these studies for a number of possible reasons: 1) NK cell activation likely differs during mCMV and γHV68 infection 2) these activities of NK cells may be modulated by the virus dose or strain and 3) control of WNV might be mediated differently compared to FV since WNV replicates in neurons [34] whereas FV replicates in erythrocyte precursor cells and B cells. To our knowledge NK cell responses have not been characterized in persistently γHV68 infected mice so far.

Recently Mekker et al. published that LCMV clearance in mice was not influenced by persistent mCMV infection [32]. In their mCMV infection model the virus was cleared at 6–8 weeks post infection whereas we could detect replicating mCMV in salivary glands at least up to 12 weeks post mCMV infection (Additional file 1: Figure S1) suggesting that replicating mCMV might be necessary to influence anti-viral immune responses against a secondary infection. Additionally Mekker et al. used the mCMV Δm157 virus, which does not induce a strong NK cell response in C57BL/6 mice. Instead, we used the mCMV Smith strain in C57BL/6 mice, which was shown to induce strong NK cell response that is responsible for the early control of the mCMV infection [23]. The mCMV specific m157 protein binds to the NK cell receptor Ly49H and induces strong NK cell activation and proliferation. This interaction combined with long-term virus replication might be necessary to generate effector and/or memory NK cell responses, which can improve anti-viral immune responses [35].

Our data suggests that mCMV-primed NK cells increased the magnitude of the FV-specific CD8 T cell response. At first glance our findings seem to be contradictory to recently published studies showing a negative effect of NK cells on the priming of virus-specific CD8 T cell responses [36-38]. However, in these studies the impact of NK cells on the outcome of a primary virus infection was examined in previously naïve mice. In our study, we demonstrated the positive impact of previously activated, memory-type NK cells in mice persistently infected with mCMV on the priming of retrovirus-specific CD8 T cells. In high-risk HIV-exposed seronegative individuals, an increased NK cell activity has been found, indicating that NK cells might also be involved in the resistance against primary HIV-1 infection [39,40].

Our current data also seem to contradict previous studies on the interaction of CMV and retroviruses in HIV infected patients. These studies demonstrate reactivation of CMV in the late stage of HIV infection, when patients have low CD4 T cell counts [2-5] and a drop in CMV-specific CD4 T cell numbers [6]. Under these circumstances CMV seropositivity can clearly be harmful for a retrovirus infected host. However, here we studied a primary retrovirus infection in a CMV seropositive host, a scenario that has not been studied so far in HIV infected patients. These contradictory findings indicate that on one hand CMV seropositivity might be beneficial during a primary retrovirus infection on the other hand it becomes detrimental during a chronic retrovirus infection.

## Conclusion

Taken together, mCMV primed NK cells that produce IFNγ were able to promote the FV-specific CD8 T cell response after FV superinfection. Our results suggest that activated NK cells in CMV seropositive individuals might also contribute to the control of primary HIV infection. There are several studies on primary HIV infection but none of them analyzes the CMV status of these patients.

## Methods

### Mice

Six to eight weeks old C57BL/6 (H-2b) male mice were obtained from Harlan (Horst, Netherlands). The mice were infected i.p. with 1 × 10^5^ PFU mCMV (Smith strain) or were inoculated with PBS as control. The mCMV stock was prepared from salivary glands of Balb/c mice 15 days post infection [41]. During the persistent phase (5–10 weeks post infection), persistently mCMV and naive mice (controls) were infected i.v. with 20,000 spleen focus-forming units (SFFU) of Friend virus (FV). The FV stock was obtained from 15% spleen cell homogenates from BALB/c mice infected 14 days previously with 3,000 SFFU. The FV stock was not contaminated by lactate dehydrogenase-elevating virus (LDV) [42]. All virus stocks were diluted with PBS. Mice were maintained under pathogen-free conditions and treated in according with the regulations and guidelines of institutional animal care of the University Duisburg-Essen, Germany.

### Virus titer detection

Titrations of single-cell suspensions of FV infected splenocytes were performed on susceptible *Mus dunni* cells, co-cultivated for 3 days and stained with F-MuLV envelope-specific monoclonal antibody 720 as previously described [43]. Results were detected as log_10_ infectious centers (IC) per spleen.

The number of mCMV PFU was determined by plaque assay using a 10% homogenate of tissue taken from individual mice and tenfold dilutions of this homogenate on mouse embryonic fibroblasts (GTKO) cells were used to quantify mCMV titers [44]. Titers reported are numbers of log_10_ PFU per whole liver and salivary gland.

### Cell-surface and intracellular staining by flow cytometry

Cell-surface staining was performed with the following antibodies: anti-CD3 (17A2), anti-CD49b (DX5), anti-CD69 (H1.2 F3), anti-NK1.1 (PK136), anti-CD43 (1B11), anti-CD4 (RM4-5), anti-CD8 (53–6.7), anti-CD107a (1D4B), anti-Ter119 (TER-119), anti-KLRG-1 (2 F1), anti-CD44 (IM7), anti-CD19 (MB19-1), anti-B220 (RA3-6B2) and anti-CD62L (MEL-14).

Dead cells (positive for fixable viable dye; eBioscience) were excluded from analysis. Data were acquired on a LSR II flow cytometer (BD Biosciences) and analyses were performed using FACSDiva (BD Biosciences) and Flow Jo (Tree Star) software.

For intracellular staining of IFNγ in NK cells, up to 4 × 10^6^ splenocytes were stimulated with PMA (400 ng/ml) and ionomycin (500 ng/ml) in the presence of brefeldin A for 3 h at 37°C. Stimulated cells were pre-incubated with an Fc Block (2.4G2) in FACS buffer (HBBS, 2% FCS, 0.1% NaN_3_) and stained for 20 min at 4°C with various combinations of fluorescently tagged monoclonal antibodies. After washing, cells were permeabilized using BD Cytofix/Cytoperm solution and then stained in BD Permwash using monoclonal antibodies specific for various cytokines. Intracellular staining of granzmye B (clone GB12) and Foxp3 was performed without *in vitro* stimulation. Foxp3 expression was detected using the Foxp3 antibody (clone FJK-16 s) and the Foxp3 staining kit (eBioscience).

### Tetramers and tetramer staining

For quantification of virus-specific CD8 T cells, spleen cells were stained with anti-CD8, anti-CD43 and MHC class I H2-D^b^ tetramers specific for the FV GagL epitope [45,46] for 30 min at room temperature as described [47].

### *In vivo* cytotoxicity assay

The *in vivo* CTL assay was performed as described previously [47]. Briefly, splenocytes from naïve CD45.1^+^ C57BL/6 mice were loaded with 1 μM D^b^GagL peptide [45,46]. These loaded splenocytes were stained with 200 nM 5-(and 6-) carboxyfluorescein diacetate succinimidyl ester (CFSE; Molecular Probes). As control, spleen cells without peptide and no CSFE labeling were used. Peptide loaded and non-loaded splenocytes were mixed 1:1 and transferred i.v. (10^7^ cells of each population) into FV-infected CD45.2^+^ C56BL/6 mice at day 8 post FV infection. Two hours after the transfer of cells, splenocytes were harvested, and cell suspensions were prepared. Target cells were distinguished from recipient cells by the surface molecules CD45.1 and CD45.1 and peptide loaded and non-loaded cells by CFSE staining. The percentage of killing was calculated as follows: 100 − ([(% peptide pulsed in infected/% unpulsed in infected)/(% peptide pulsed in uninfected/% unpulsed in uninfected)] × 100).

### Antibody treatment

NK1.1^+^ cell depletion was performed using 0.5 ml of supernatant fluid containing NK1.1-specific monoclonal antibody PK136 [48]. Four days after the last NK1.1 injection over 90% off all NK cells (DX5^+^, NK1.1^+^, NKp46^+^, CD3^-^) were depleted in naïve and persistently mCMV infected mice. Additionally we detected an up to 89% depletion of NKT cells (NK1.1^+^, NKp46^+^, CD3^-^) in naïve and persistently mCMV infected mice at this time-point. No depletion of γδT and CD8 T cells was detected after NK1.1 antibody injection. Neutralization of IFNγ was done by injection of 150 to 250 μg XMG1.2 antibody in 0.2 ml PBS (Bio-X-Cell). Antibodies were injected i.p. into mice at day −1, 2 and 4 post FV infection.

### Cytokine assay

Mouse IFNγ levels were detected using the Femto-HS Elisa kit (eBioscience) according to the manufacturers’ instructions.

### Ethics statement

Animal experiments were performed in strict accordance with the German regulations of the Society for Laboratory Animal Science (GV-SOLAS) and the European Health Law of the Federation of Laboratory Animal Science Associations (FELASA). The protocol was approved by the North Rhine-Westphalia State Agency for Nature, Environment and Consumer Protection (LANUF). All efforts were made to minimize suffering.

## Competing interests

The authors declare that they have no competing interests.

## Authors’ contributions

SF, JP, TS, JD carried out experiments and analyzed the data; KG, UD analyzed data; ARMK and UD wrote the manuscript. All authors read and approved the final manuscript.

## Supplementary Material

Additional file 1: Figure S1 Replication of persistent mCMV. MCMV loads were analyzed at 5–10 weeks post mCMV infection in livers and salivary glands. Data are pooled from 4 independent experiments with 16–19 mice per group. Click here for file

Additional file 2: Figure S2 NK cell depletion or IFNγ neutralization had no direct effect on viral loads or T cell responses in FV infection. Naïve mice were infected with FV and a,c) viral loads and b,d) granzymeB^+^ FV tetramer^+^ T cells were determined in untreated (open circles, white bars) and anti-NK1.1 or anti-IFNγ antibody (half-filled circles, stripped bars) treated mice at day 8 post FV infection. Data represent the mean of two individual experiments with 4–7 mice/group. Click here for file
