# Supplementary figures and images for: NK cells improve control of friend virus infection in mice persistently infected with murine cytomegalovirus
Source: Retrovirology. 2013 Jun 5;10:58. doi: 10.1186/1742-4690-10-58 (PMC3744174; doi:10.1186/1742-4690-10-58)

## Slide: page1
Additional file 1: Figure S1
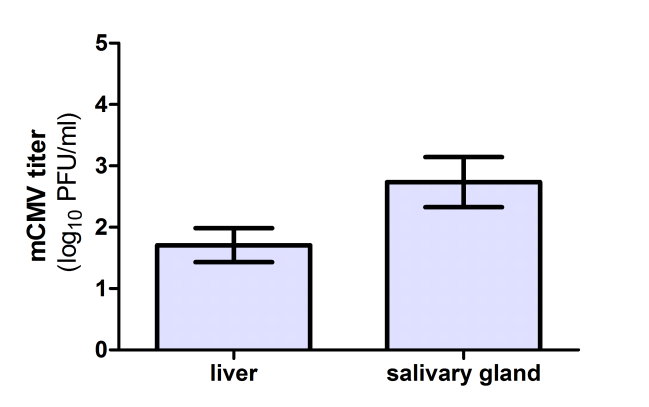

Supplement: Additional file 1: Figure S1 — Replication of persistent mCMV. MCMV loads were analyzed at 5–10 weeks post mCMV infection in livers and salivary glands. Data are pooled from 4 independent experiments with 16–19 mice per group. [file 1742-4690-10-58-S1.odp]

## Slide: page1
Additional file 2: Figure S2
a)
b)
c)
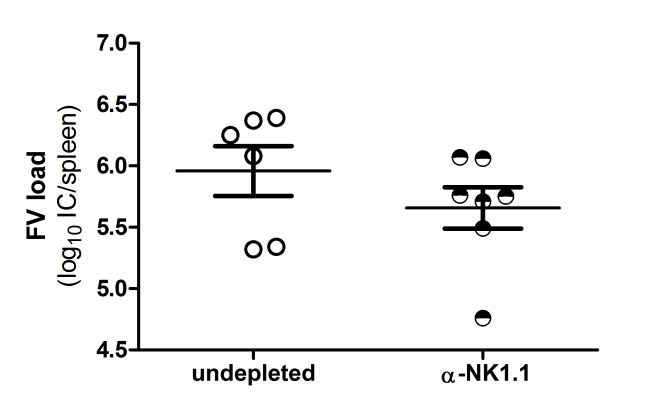

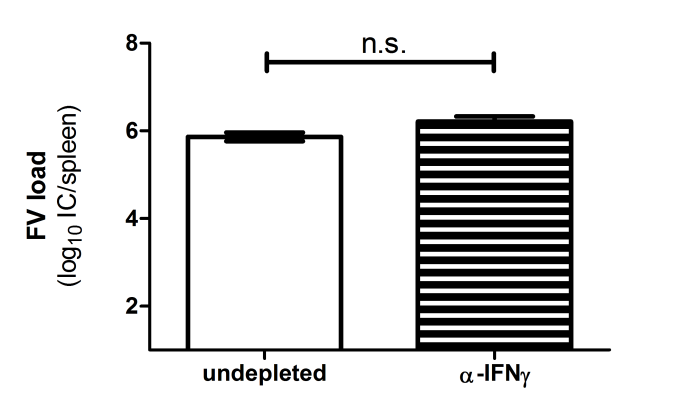

d)
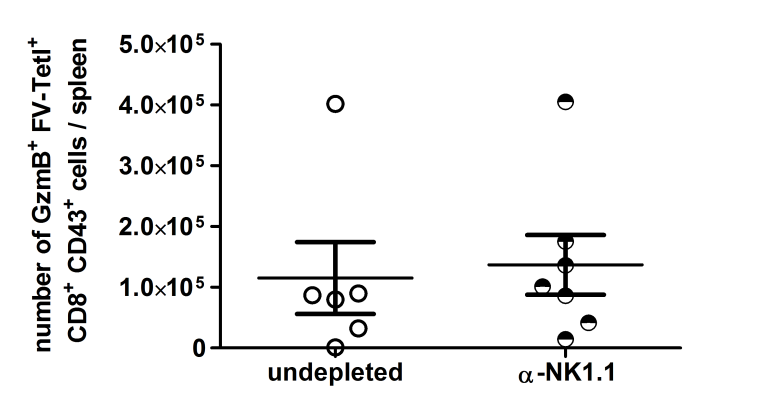

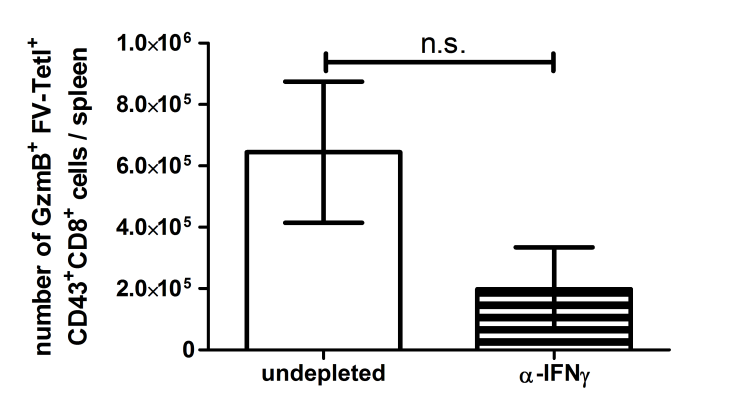

Supplement: Additional file 2: Figure S2 — NK cell depletion or IFNγ neutralization had no direct effect on viral loads or T cell responses in FV infection. Naïve mice were infected with FV and a,c) viral loads and b,d) granzymeB+ FV tetramer+ T cells were determined in untreated (open circles, white bars) and anti-NK1.1 or anti-IFNγ antibody (half-filled circles, stripped bars) treated mice at day 8 post FV infection. Data represent the mean of two individual experiments with 4–7 mice/group. [file 1742-4690-10-58-S2.odp]
